# Widely Targeted Metabolomics Analysis of the Roots, Stems, Leaves, Flowers, and Fruits of *Camellia luteoflora*, a Species with an Extremely Small Population

**DOI:** 10.3390/molecules29194754

**Published:** 2024-10-08

**Authors:** Weicheng Yang, Fen Liu, Gaoyin Wu, Sheng Liang, Xiaojie Bai, Bangyou Liu, Bingcheng Zhang, Hangdan Chen, Jiao Yang

**Affiliations:** 1 School of Life Sciences, Guizhou Normal University, Guiyang 550025, China; yangweicheng0908@sina.com (W.Y.); 15117707714@163.com (F.L.); zbc010811@163.com (B.Z.); chd0324@163.com (H.C.); 13096720857@163.com (J.Y.); 2 Chishui Alsophila National Nature Reserve Management Bureau, Chishui 646259, China; 17785964995@163.com (S.L.); 17385115856@163.com (X.B.); 17384197633@163.com (B.L.)

**Keywords:** *Camellia luteoflora*, different organs, chemical constituents, differentially expressed metabolites

## Abstract

*Camellia luteoflora* is a rare and endangered plant endemic to China. It has high ornamental and potential economic and medicinal value, and is an important germplasm resource of Camellia. To understand the distributions and differences in metabolites from different parts of *C. luteoflora*, in this study, we used liquid chromatography–tandem mass spectrometry (LC–MS/MS) to examine the types and contents of chemical constituents in five organs of *C. luteoflora*: roots, stems, leaves, flowers, and fruits. The results showed that a total of 815 metabolites were identified in the five organs and were classified into 18 main categories, including terpenoids (17.1%), amino acids (10.4%), flavonoids (10.3%), sugars and alcohols (9.8%), organic acids (9.0%), lipids (7.1%), polyphenols (4.8%), alkaloids (4.8%), etc. A total of 684 differentially expressed metabolites (DEMs) in five organs were obtained and annotated into 217 KEGG metabolic pathways, among which metabolic pathways, ABC transporters, the biosynthesis of cofactors, and the biosynthesis of amino acids were significantly enriched. In DEMs, flowers are rich in flavonoids, polyphenols, organic acids, and steroids; fruits are rich in amino acids, alkaloids, vitamins, and xanthones; stems are rich in lignans; and leaves have the highest relative content of phenylpropanoids, ketoaldehydic acids, quinones, sugars and alcohols, terpenoids, coumarins, lipids, and others; meanwhile, the metabolite content is lower in roots. Among the dominant DEMs, 58 were in roots, including arachidonic acid, lucidone, isoliquiritigenin, etc.; 75 were in flowers, including mannose, shikimic acid, d-gluconic acid, kaempferol, etc.; 45 were in the fruit, including pterostilbene, l-ascorbic acid, riboflavin, etc.; 27 were in the stems, including salicylic acid, d-(-)-quinic acid, mannitol, (-)-catechin gallate, etc.; there was a maximum number of 119 dominant metabolites in the leaves, including oleanolic acid, l-glucose, d-arabitol, eugenol, etc. In sum, the rich chemical composition of *C. luteoflora* and the significant differences in the relative contents of metabolites in different organs will provide theoretical references for the study of tea, flower tea, edible oil, nutraceuticals, and the medicinal components of *C. luteoflora*.

## 1. Introduction

*Camellia luteoflora* is a genus of *Camellia* in the Theaceae family. It is a perennial evergreen shrub or small tree and is an endemic plant of the China–Himalaya subregion. It was first discovered in November 1981 in Jinsha Gully, Chishui city, with an extremely small population in Guizhou Province, China, where it is an endemic, rare, and endangered plant [1]. From a phylogenetic point of view, *C. luteoflora* is one of the more primitive populations in the genus *Camellia* and is considered to be an ancient relict species [2] that is mainly distributed in Chishui city, Guizhou Province, and in Gulin County and Changning County, Sichuan Province [3]. It grows in an understory habitat that is warm and humid, and adjacent to water sources. However, the high canopy density and weak light conditions under the forest greatly affect the growth, breeding, and natural regeneration of *C. luteoflora* [4], while Bai et al. [5] found that *C. luteoflora* has the largest important value and niche width value in the community, which has important ecological value for the protection of endangered species and community stability. In addition, China is rich in camellia resources. According to statistics, there are more than 30,000 registered camellia varieties, and *C. luteoflora* with rare golden yellow genes has a smaller flower type and a clear scent, and the leaves are waxy, glossy, wide, and thick, with high ornamental value, known as the “Queen of Camellias” [6,7]. Like other *Camellia* species, these plants are rich in bioactive substances such as flavonoids, terpenoids, polyphenols, phytosterols, fatty acids, and alkaloids. These compounds hold significant pharmacological potential [8,9], including for anti-oxidation, anti-aging, neuroprotection, anti-osteoporosis, skin repair, and immunity enhancement, as well as the prevention and improvement of various diseases [10,11]. In 2022, the *No. 1 document* of the Central Committee of the Communist Party of China and the State Council on the key work of comprehensively promoting rural revitalization was released. The document clearly points out that it is necessary to support the expansion of the planting area of *C. oleifera* and the transformation and upgrading of low-yield forests. By 2025, the expected planting area of *C. oleifera* will reach 6 × 10^4^ km^2^, and the output of tea oil will reach 2 × 10^9^ kg, double that of 2022. Secondly, tea is the most popular flavor and functional plant beverage, and the consumer population accounts for two-thirds of the world’s total population. Tea is classified into six categories based on processing procedures such as green tea, white tea, yellow tea, oolong tea, black tea, and dark tea [12]; however, developing new quality tea products can stimulate and improve the market position of existing tea products. At present, the famous scented teas that occupy a certain position in China’s scented tea market include jasmine tea, chrysanthemum tea, honeysuckle tea, osmanthus tea, rose tea, etc. Their industry has developed well and has broad prospects for future development. Because *C. nitidissima* can emit a pleasant fragrance, its scented tea has a strong tea flavor and a floral aroma, which is widely popular among consumers. In recent years, it has been strongly sought after by the market, which has led to its prices remaining high for years [13]. These show that *Camellia* plants have significant economic, ecological, and social benefits in the fields of agriculture, forestry, food, medicine, cosmetics, and other industries.

Due to the scarcity of wild resources and the narrow distribution area of *C. luteoflora*, as well as the influence of natural factors such as human interference, *Exobasidium gracile* (shirai) Syd, and witches’ broom disease [14], coupled with certain reproductive barriers in seeds [15], the difficulty of population self-renewal, interference pressure, and the obvious trend of population decline [5], the species is facing endangerment and possible extinction;. it has been classified as “critically endangered” on the Red List of Threatened Species of the International Union for Conservation of Nature (https://www.iucnredlist.org/species/62055668/62055674 (accessed on 23 May 2024)), resulting in basic research of *C. luteoflora* relatively lagging behind, and its development and utilization are limited. At present, the research of *C. luteoflora* mainly focuses on population structure [3], community ecology [16,17], habitat suitability [18], chloroplast genome [19], and cutting propagation technology [20]. Jin et al. [21] used GC–MS to detect the volatile components of *C. luteoflora*, and identified 74 and 72 compounds in flowers and leaves, respectively. There are many kinds of aliphatic compounds and terpenoids: the highest content in flowers is linalool (16.71%) and the highest content in leaves was trans-2-hexenal (20.63%). Liu et al. [22] used high-performance liquid chromatography (HPLC) to analyze the amino acid and main fatty acid components of five wild *Camellia* seeds in Guizhou, and revealed that *C. luteoflora* had a higher essential amino acid content. However, the non-volatile components and contents of flowers, leaves, roots, stems, and fruits (except amino acids and fatty acids) have not been reported. Therefore, it is necessary to quantitatively analyze its components and differences. Metabolomics is a technique for the large-scale detection of all metabolites in biological systems, such as entire organisms, tissues, or individual cells, which includes targeted metabolomics, non-targeted metabolomics, and widely targeted metabolomics. Widely targeted metabolomics analysis is a new method that combines the advantages of non-targeted and targeted metabolomics. It has the characteristics of high throughput, super sensitivity, wide coverage, and accurate qualitative and quantitative analysis. In this study, widely targeted metabolomics was used to identify and analyze the chemical constituents of the roots, stems, leaves, flowers, and fruits of *C. luteoflora*, and to study their diversity and enrichment characteristics in different organs, so as to provide a theoretical basis and data support for the accurate excavation and utilization of chemical constituents of *C. luteoflora*. At the same time, the research results have important guiding significance for the subsequent research on tea oil, scented tea, leaf tea, health care products, and pharmaceutical preparations.

## 2. Results

### 2.1. Multivariate Statistical Analysis

**Figure 1:**
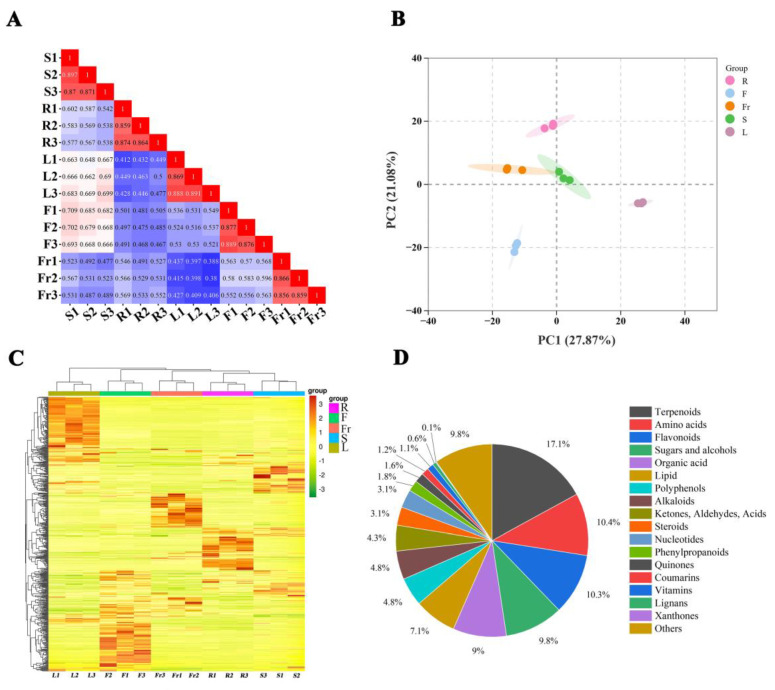
PCA and metabolite accumulation of different organs of *C. luteoflora*. (**A**) Correlation plot between samples; (**B**) PCA score plot of all samples; (**C**) cluster heat map of metabolites; (**D**) classification of metabolites.

To evaluate the metabolite mass spectrometry data for the 15 samples, correlation analysis was performed between the samples to assess the biological replication between samples within the group. Correlation analysis of the 15 samples revealed that the correlation coefficients within each group were >0.85 (Figure 1A), indicating good experimental repeatability. A comprehensive analysis of the samples using unsupervised PCA showed that the first principal component explained 27.87%, the second principal component explained 21.08% of the variance, and the samples clustered within the different organ groups were clearly separated in these two dimensions (Figure 1B), suggesting that there is considerable variation in the metabolites of the roots, stems, leaves, flowers, and fruits of *C. luteoflora*. The HCA of metabolites from different organs of *C. luteoflora* was performed by clustering heatmaps, indicating that the metabolite profiles of roots, stems, leaves, flowers, and fruits of *C. luteoflora* were clearly divided into five clusters, with significant differences in the relative abundance of metabolites from different organs (Figure 1C). The metabolites of different organs of *C. luteoflora* were analyzed by the OPLS-DA model. The metabolites of different organs were significantly separated, and all of them were within the confidence intervals, indicating that the metabolites of different organs of *C. luteoflora* were significantly different and that Q2Y > 0.9 for all controls (Supplementary Materials), which indicated that the OPLS-DA model was stable and reliable.

### 2.2. Metabolite Analysis of the Five Studied Organs of C. luteoflora

A total of 815 metabolites were identified in the five organs of *C. luteoflora* (Supplementary Materials) and were categorized into 18 classes: 139 terpenoids (17.1%), 85 amino acids (10.4%), 84 flavonoids (10.3%), 80 sugars and alcohols (9.8%), 73 organic acids (9.0%), 58 lipids (7.1%), 39 polyphenols (4.8%), 39 alkaloids (4.8%), 35 ketones, aldehydes, acids (4.3%), 25 steroids (3.1%), 25 nucleotides (3.1%), 15 phenylpropanoids (1.8%), 13 quinones (1.6%), 10 coumarins, 9 vitamins, 5 lignans, 1 xanthone, and 80 others (9.8%) (Figure 1D).

### 2.3. Analysis of the Relative Contents of Metabolites in the Different Organs of C. luteoflora

The relative contents of metabolites in the roots, flowers, fruits, stems, and leaves of *C. luteoflora* were significantly different (Table 1). The contents of flavonoids, polyphenols, organic acids, and steroids were significantly greater in the flowers than in the other four organs, and there was no difference in the content of nucleotides between the flowers and fruits, which was significantly greater than that in the roots, stems, and leaves. The fruits contained high levels of amino acids, alkaloids, and vitamins. It is worth noting that among the five organs, only the fruits contained xanthones; lignans were the most abundant in the stems, with no significant difference from the fruits and leaves, and were the least abundant in the flowers; phenylpropanoids, ketones, aldehydes, acids, quinones, sugars and alcohols, terpenoids, coumarins, lipids, and others were the most abundant in the leaves, whereas the contents of all 18 types of metabolites in the roots were lower than those in the other four organs.

### 2.4. DEM Analysis in the Different Organs of C. luteoflora

**Figure 2:**
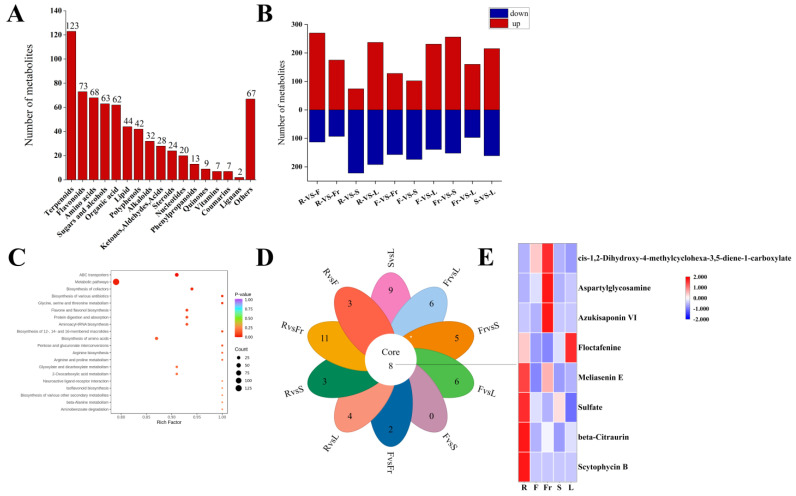
Analysis of DEMs in five organs of *C. luteoflora*. (**A**) The number of different types of DEMs in five organs; (**B**) the number of up-regulated and down-regulated DEMs between R, F, Fr, S, and L, and the red column represents the up-regulated DEMs; blue column represents down-regulated DEMs; (**C**) the top 20 KEGG metabolic pathways of DEMs were significantly enriched; (**D**) petal diagram of DEMs; (**E**) the heat map of common metabolites.

A total of 684 DEMs (Supplementary Materials) were screened from the roots, stems, leaves, flowers, and fruits of *C. luteoflora* according to FC > 1, *P* < 0.05, and VIP ≥ 1. Except xanthones, they were divided into 17 categories (Figure 2A), including 123 terpenoids, 73 flavonoids, 68 amino acids, 63 sugars and alcohols, 62 organic acids, 44 lipids, 42 polyphenols, 32 alkaloids, 28 ketones, aldehydes acids, 24 steroids, 20 nucleotides, 13 phenylpropanoids, 9 quinones, 7 vitamins, 7 coumarins, 2 lignans, and 67 others. Among them, 36 terpenoids were enriched in the roots; 13 lipids and 1 lignan were enriched in the stems; 19 amino acids, 26 sugars and alcohols, 30 organic acids, 12 polyphenols, 8 nucleotides, 9 ketones, aldehydes acids were enriched in the flowers; 20 amino acids, 6 steroids, and 2 vitamins were enriched in the fruits; 47 terpenoids, 25 flavonoids, 9 alkaloids, 24 others, 14 polyphenols, 5 phenylpropanoids, 4 quinones, and 4 coumarins were enriched in the leaves (Supplementary Materials).

Compared with the roots, there were 370 DEMs (231 up-regulated and 139 down-regulated), 276 DEMs (102 up-regulated and 174 down-regulated), 257 DEMs (160 up-regulated and 97 down-regulated), and 408 DEMs (256 up-regulated and 152 down-regulated) in the flowers, fruits, stems and leaves, respectively. Compared with the flowers, there were 296 DEMs (74 up-regulated and 222 down-regulated), 285 DEMs (128 up-regulated and 157 down-regulated), and 429 DEMs (237 up-regulated and 192 down-regulated) in the fruits, stems, and leaves, respectively. Compared with the fruits, there were 268 DEMs (175 up-regulated and 93 down-regulated) and 383 DEMs (270 up-regulated and 113 down-regulated) in the stems and leaves, respectively; there were 376 DEMs (215 up-regulated and 161 down-regulated) in the stems and leaves (Supplementary Materials).

A total of 684 DEMs were annotated into 217 KEGG metabolic pathways, among which ABC transporters, metabolic pathways, the biosynthesis of cofactors, and the biosynthesis of amino acids were significantly enriched.

Pairwise comparison of DEMs in five organs revealed that there were eight common DEMs obtained in the 10 groups, including four terpenoids (Scytophycin B, Azukisaponin VI, Meliasenin E, beta-Citraurin), two organic acids (Floctafenine, cis-1,2-Dihydroxy-4-methylcyclohexa-3,5-diene-1-carboxylate), one amino acid (Aspartylglycosamine), and one other (Sulfate).

### 2.5. Analysis of Dominant DEMs in the Different Organs of C. luteoflora

**Figure 3:**
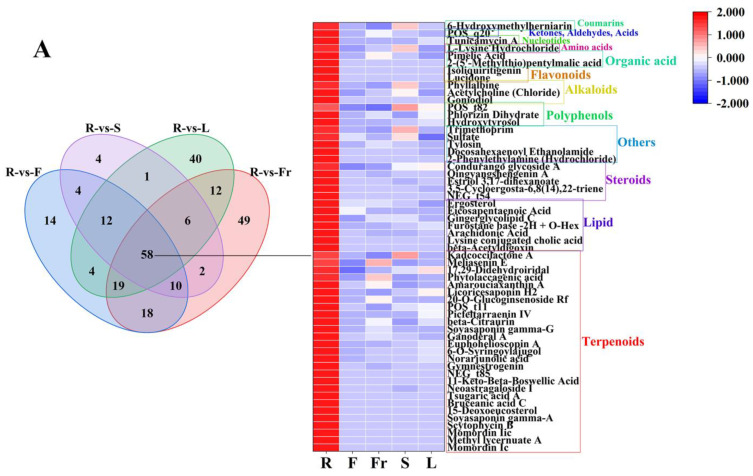

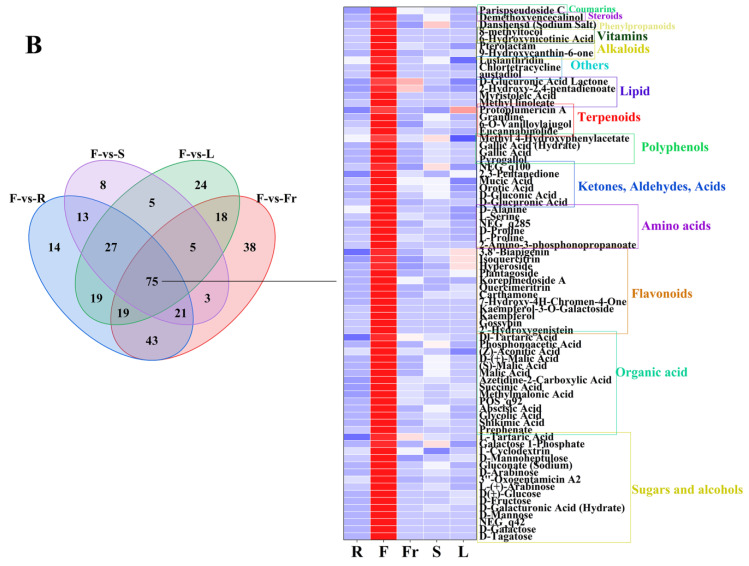

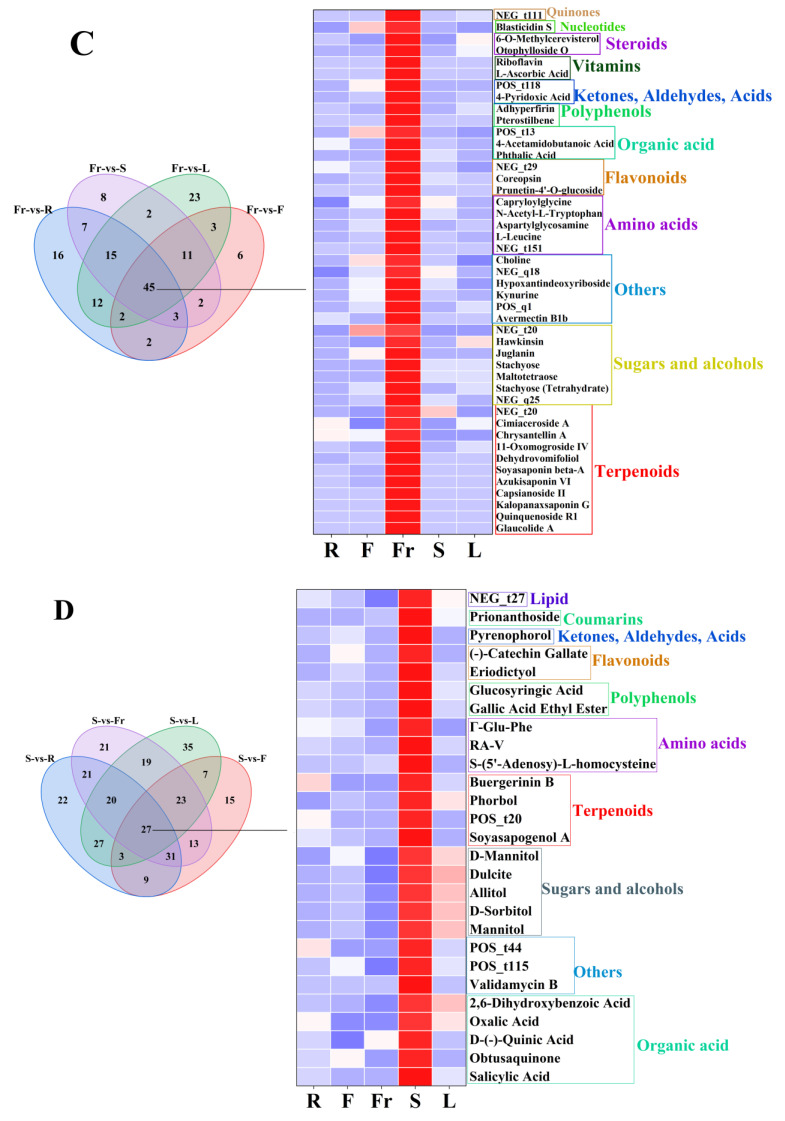

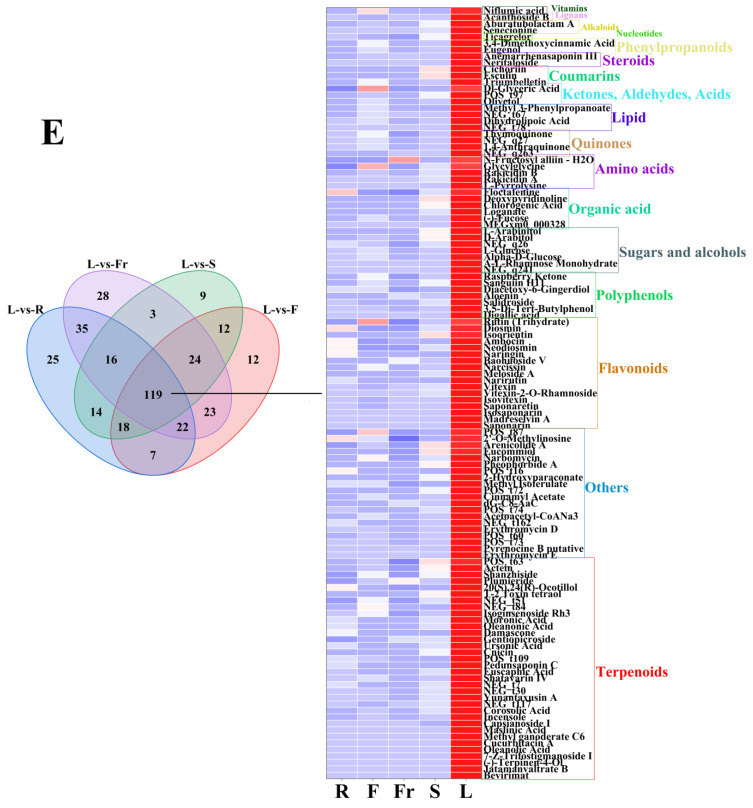
The dominant DEMs of roots, flowers, fruits, stems, and leaves of *C. luteoflora*. (**A**) The dominant DEMs of R; (**B**) The dominant DEMs of F; (**C**) The dominant DEMs of Fr; (**D**) The dominant DEMs of S; (**E**) The dominant DEMs of L.

FC < 1, *P* < 0.05, and VIP ≥ 1 were used as the threshold of dominant DEMs for analysis and screening, and 58, 75, 45, 27, and 119 dominant metabolites were obtained in roots, flowers, fruits, stems, and leaves, respectively (Figure 3).

The dominant DEMs of R, F, Fr, S, and L were classified by the HMDB database. According to the classification diagram of dominant DEMs, the dominant DEMs of R were 27 terpenoids (Momordin Ic, Gymnestrogenin, Momordin Iic, Ganoderal A, beta-Citraurin, etc.), 7 lipids (beta-Acetyldigoxin, Ergosterol, arachidonic acid, eicosapentaenoic acid, Gingerglycolipid C, etc.), 5 steroids (3,5-Cycloergosta-6,8(14),22-triene, Estriol 3,17-dihexanoate, Qingyangshengenin A, Condurango glycoside A, Qingyangshengenin A), 5 others (2-Phenylethylamine (Hydrochloride), Docosahexaenoyl Ethanolamide, Tylosin, Sulfate, Trimethoprim), 3 polyphenols (Hydroxytyrosol, Phlorizin Dihydrate, p-Vinylphenyl O-[beta-D-apiofuranosyl-(1-6)]-beta-D-glucopyranoside), 3 alkaloids (Goniodiol, Acetylcholine (Chloride), Phyllalbine), 2 flavonoids (Lucidone, Isoliquiritigenin), 2 organic acids (2-(5′-Methylthio)pentylmalic acid, Pimelic Acid), 1 amino acid (L-Lysine Hydrochloride), 1 nucleotide (Tunicamycin A), 1 ketone, aldehyde, acid ((3-Carboxypropyl)Trimethylammonium Chloride), and 1 coumarin (6-Hydroxymethylherniarin).

The dominant DEMs of F were 15 sugars and alcohols (D-Tagatose, D-Galactose, D-Mannose, D-Mannoheptulose, D-Fructose, etc.), 14 organic acids (prephenate, shikimic acid, succinic acid, phosphonoacetic acid, malic acid, etc.), 12 flavonoids (2′-Hydroxygenistein, Gossypin, kaempferol, Kaempferol-3-O-Galactoside, Isoquercitrin, etc.), 6 amino acids (2-Amino-3-phosphonopropanoate, L-Proline, D-Proline, L-Serine, D-Alanine, etc.), 6 ketones, aldehydes, acids (dD-Glucuronic Acid, D-gluconic Acid, Orotic Acid, Mucic Acid, 2,3-Pentanedione, etc.), 4 polyphenols (Pyrogallol, Gallic Acid, Gallic Acid (Hydrate), Methyl 4-Hydroxyphenylacetate), 4 terpenoids (Eucannabinolide, 6-O-Vanilloylajugol, Graniline, Protoplumericin A), 4 lipids (Methyl linoleate, Myristoleic Acid, 2-Hydroxy-2,4-pentadienoate, D-Glucuronic Acid Lactone), 3 others (austadiol, Chlortetracycline, Lusianthridin), 3 alkaloids (9-Hydroxycanthin-6-one, Pterolactam, 6-Hydroxynicotinic Acid), 1 vitamin (8-methyltocol), 1 phenylpropanoid (Danshensu (Sodium Salt)), 1 steroid (Parispseudoside C), and 1 coumarin (Demethoxyencecalinol).

The dominant DEMs of Fr were 11 terpenoids (Glaucolide A, Quinquenoside R1, Dehydrovomifoliol, Capsianoside II, Azukisaponin VI, etc.), 7 sugars and alcohols (Stachyose (Tetrahydrate), Maltotetraose, Stachyose, 2′″-N-Acetyl-6′″-Deamino-6′″-Hydroxyparomomycin Ii, Hawkinsin, etc.), 6 others (Avermectin B1b, 1,4-Diaminobutane (Dihydrochloride), Kynurine, Hypoxantindeoxyriboside, Choline, etc.), 5 amino acids ([D-Asp3,E-Dhb7]-Microcystin-RR, L-Leucine, Aspartylglycosamine, N-Acetyl-L-Tryptophan, Capryloylglycine), 3 flavonoids (Prunetin-4′-O-glucoside, Coreopsin, Kaempferol-3-Rhamnoside-4″-Rhamnoside-7-Rhamnoside), 3 organic acids (Phthalic Acid, 4-Acetamidobutanoic Acid, cis-1,2-Dihydroxy-4-methylcyclohexa-3,5-diene-1-carboxylate), 2 polyphenols (pterostilbene, Adhyperfirin), 2 ketones, aldehydes, acids (4-Pyridoxic Acid, methyl 3,4,5-trihydroxycyclohexene-1-carboxylate), 2 vitamins (L-ascorbic acid, riboflavin), 2 steroids (Otophylloside O, 6-O-Methylcerevisterol), 1 nucleotide (Blasticidin S), and 1 quinone (Chrysophanol 1-O-beta-tetraglucoside).

The dominant DEMs of S were five organic acids (Salicylic Acid, Obtusaquinone, D-(-)-quinic acid, Oxalic Acid, 2,6-Dihydroxybenzoic Acid), four sugars and alcohols (D-Sorbitol, Allitol, Dulcite, D-Mannitol, Mannitol), four terpenoids (Soyasapogenol ANCGC00384973-01_C13H22O4_(3E)-4-(1,2,4-Trihydroxy-2,6,6-trimethylcyclohexyl)-3-buten-2-one, Phorbol, Buergerinin B), four others (Validamycin B, 4-(2-methyl-6-oxopyran-3-yl)butanoic acid, Dereplicator Identification—E’Surugamide_D), three amino acids (S-(5′-Adenosy)-L-homocysteine, RA-V, Γ-Glu-Phe), 2 polyphenols(Gallic Acid Ethyl Ester, Glucosyringic Acid), two flavonoids (Eriodictyol, (-)-catechin gallate), one ketone, aldehyde, acid (Pyrenophorol), one coumarin (Prionanthoside), and one lipid (NCGC00380867-01_C27H46O9_9,12,15-Octadecatrienoic acid, 3-(hexopyranosyloxy)-2-hydroxypropyl ester, (9Z,12Z,15Z)-).

**Figure 4:**
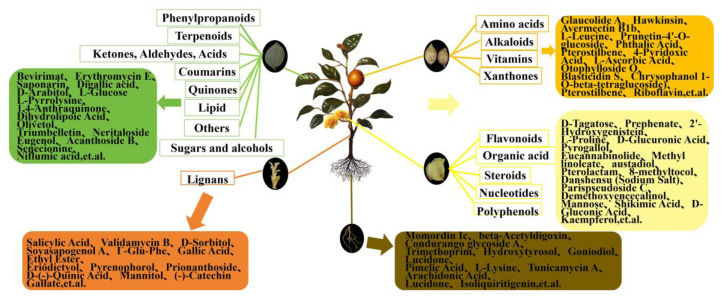
Summary of total metabolite enrichment and some dominant DEMs in roots, flowers, fruits, stems, and leaves of *C. luteoflora*. The rectangles represent the enrichment of the relative total content of various metabolites in different organs, and the rounded rectangles represent the enrichment of the relative content of some dominant DEMs.

The dominant DEMs of L were 34 terpenoids (Bevirimat, Cnicin, (-)-Terpinen-4-Ol, Actein, oleanolic acid, etc.), 20 others (Erythromycin E, Pyrenocine B putative, Erythromycin D, Acetoacetyl-CoANa3, dG-C8-AaC, etc.), 17 flavonoids (Saponarin, Narirutin, Naringin, Diosmin, Isovitexin, etc.), 7 polyphenols (Digallic acid, Raspberry Ketone, Salidroside, Aloenin, Diacetoxy-6-Gingerdiol, etc.), 7 sugars and alcohols (D-Arabitol, A-L-Rhamnose Monohydrate, Alpha-D-Glucose, L-Glucose, L-Arabinitol, etc.), 6 organic acids Floctafenine, MEGxm0_000328, Loganate, Chlorogenic Acid, etc.), 5 amino acids (L-Pyrrolysine, Rakicidin A, Rakicidin B, Glycylglycine, N-Fructosyl alliin—H_2_O), 4 quinones(Physcion 8-O-Β-D-Glucopyranoside, 1,4-Anthraquinone, 2-Tert-Butyl-1,4-Benzoquinone, Thymoquinone), 4 lipids (PG(18:3(9Z,12Z,15Z)/20:3(8Z,11Z,14Z)), Dihydrolipoic Acid, NCGC00380204-01_C26H38O6_Androsta-11,15-diene-14-carboxylic acid, 3,15,19-trihydroxy-4,4,8,12,16-pentamethyl-17-oxo-, methyl ester, Methyl 3-Phenylpropanoate), 3 ketones, aldehydes, acids (Olivetol, NCGC00380966-01_C12H16O4_6H-2-Benzopyran-6-one, 1,7,8,8a-tetrahydro-7,8-dihydroxy-3,5,7-trimethyl-, (7S,8S,8aS)-, Dl-Glyceric Acid), 3 coumarins (Triumbelletin, Esculin, Cichoriin), 2 steroids (Neritaloside, Anemarrhenasaponin III,), 2 phenylpropanoids (Eugenol, 3,4-Dimethoxycinnamic Acid), 2 alkaloids (Senecionine, Aburatubolactam A), 1 vitamin (Niflumic acid), and 1 nucleotide (Ticagrelor), and 1 lignan (Acanthoside B) (see Figure 4).

## 3. Materials and Methods

### 3.1. Plant Materials

**Figure 5:**
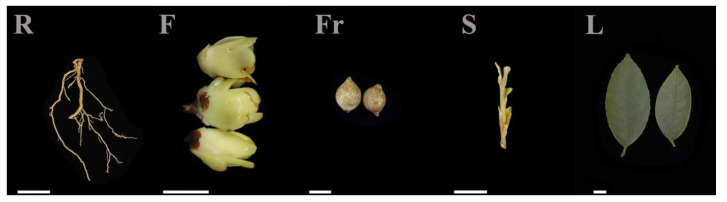
The different organs of *C. luteoflora*. R: Root; F: flower; Fr: fruit; S: stem; L: leaf. Bars = 1 cm.

Root (R), stem (S), leaf (L), and flower (F) tissues of *C. luteoflora* were collected from the China Rare Species *C. luteoflora* Reserve in Chishui City, Guizhou Province (longitude: 106°03′ E, latitude: 28°23′ N, 497 m), on November 2023, from 6 eight-year-old plants with good growth that were free of pests and disease; the fruit (Fr) was collected in December 2023 (Figure 5). The authority responsible for the *C. luteoflora* resources is the Management Bureau of Chishui *Alsophila Spinulosa* National Nature Reserve in Guizhou Province, China, who provided permission to collect the *C. luteoflora* samples. A hoe was used to randomly excavate underground roots, and the soil on the roots was washed with mineral water. Half of the opened flowers, fruits to be cracked, and semi-lignified stems and leaves from the current year were collected. The samples were quickly placed in liquid nitrogen for quick freezing, with 3 biological replicates for each sample, and stored at −80 ℃ for backup.

### 3.2. Sample Preparation

After the samples were placed in a freezer for vacuum freeze-drying (Jiaimu, Beijing, China), 50 mg of the sample was weighed and mixed with 1000 μL of methanol/acetonitrile/water = 2:2:1 (*v*:*v*:*v*) extract solution and mixed for 30 s. The samples were milled to a powder using a grinder (45 Hz, 10 min) (Bhsbio, Shanghai, China) and then incubated at −20 °C for 1 h. The samples were centrifuged at 4 °C, at 12,000 r·min^−1^ for 15 min by a low-temperature high-speed centrifuge (Eppendorf, Hamburg, Germany), 500 μL of the supernatant was removed, and the extract was dried in a vacuum concentrator. A total of 160 μL of acetonitrile/water = 1:1 (*v*:*v*) was added to the dried metabolite powder to dissolve it. The metabolite sample was vortexed for 30 s and then centrifuged at 4 °C and 12,000 r·min^−1^ for 15 min. A total of 120 μL of the supernatant was removed from the sample in a 2 mL vial and 10 μL of the supernatant of each sample was taken and mixed with the quality control (QC) samples for machine detection.

### 3.3. UPLC Conditions

The UPLC conditions were as per those described by Wang et al. [23]. An Acquity UPLC HSS T3 column (1.8 µm 2.1 × 100 mm) (Waters, Milford, MA, USA) was used and the mobile phase consisted of solvent A (0.1% formic acid and 5 mM ammonium acetate) and solvent B (acetonitrile with 0.1% formic acid, elution gradient: 0.0~1.5 min, 2% B; 5 min, 50% B; 9.0–10.0 min, 98% B; 11–14.0 min, 2% B). The flow rate was 350 μL·min^−1^ and the injection volume was 2 μL.

### 3.4. ESI–QTRAP–MS/MS Conditions

ESI–QTRAP–MS/MS were carried out as described by Wang et al. [23]. The Applied Biosystems 6500 QTRAP tandem mass spectrometry–electrospray ionization (ESI) temperature was 550 °C; ion spray voltage (IS) was 5500 V (positive ion mode)/−4500 V (negative ion mode); ion source gas I (GSI), gas II (GSII), and curtain gas (CUR) were set to 50, 55, and 35 psi, respectively; and the collision-induced ionization parameters were set to moderate. Multiple reaction monitoring (MRM) mode, collision gas (nitrogen), was set to moderate.

### 3.5. Qualitative and Quantitative Analysis of Metabolites

The qualitative and quantitative analysis of metabolites was performed using the method of Wang et al. [24]. Based on the self-built database GB-PLANT (BMK database), the qualitative analysis of substances was carried out according to the secondary spectrum information. Isotopic signals, repetitive signals containing K^+^ ions, Na^+^ ions and NH_4_^+^ ions, and repetitive signals of fragment ions that are themselves other larger-molecular-weight substances were removed during the analysis.

Quantitative analysis of metabolites was performed using the MRM mode of QQQ mass spectrometry. In the MRM mode, the quadrupole filters the precursor ions of the target substance and excludes the ions corresponding to other molecular weights to eliminate interference. After obtaining the metabolite profiling data for the different samples, peak area integration was performed for all the substance mass spectral peaks, and the integration was corrected for the mass spectral peaks of the same metabolite in different samples. The extraction, detection, and quantitative analysis of metabolites in samples were performed by Beijing Biomarker Biomarker Technology Co., Ltd. (www.biomarker.com.cn) (Beijing, China).

### 3.6. Statistical Analysis

The experimental samples were tested according to the self-constructed GB-PLANT database of Beijing Biomarker, Biomarker Technology Co., Ltd., and the metabolites were analyzed qualitatively and quantitatively using the MRM mode. Using multivariate statistical analysis, the samples were subjected to principal component analysis (PCA) and hierarchical clustering analysis (HCA), and the model stability reliability was predicted according to partial least squares discriminant analysis (PLS-DA) and orthogonal projections to latent structures-discriminant analysis (OPLS-DA). Metabolites with FC > 1, *P* < 0.05, and VIP ≥ 1 were selected as differentially expressed metabolites (DEMs). The KEGG database was utilized to annotate the DEMs.

## 4. Discussion

The relative metabolite levels of different plant parts represent the characteristics and distribution of overall nutrition and phytochemicals, which helps in the discovery of the most favorable plant parts for further targeted research on bioactive metabolites [25]. *C. luteoflora* comprises 18 kinds of chemical constituents, including terpenoids, amino acids, flavonoids, sugars and alcohols, organic acids, etc. Among them, terpenoids are the most abundant (17.1%) in *C. luteoflora*, which have the effects of lowering blood sugar, antitumour activity, antioxidant activity, protecting the liver, antimicrobial activity, kidney protection, and immune system regulation [9]. The relative content is the highest in the leaves Tang et al. [26] found that the accumulation of terpenoids in tea flowers is more than in young leaves. This may be due to the differences in the expression of terpenoids, metabolic synthesis pathways, and key enzymes in different plants and organs. Amino acids are the second most abundant substance (10.4%) in *C. luteoflora*. They are the basic units of protein and have special physiological functions in the body and are one of the indispensable nutrients in the organism, can regulate metabolism, and enhance immunity. Their type, content, and composition have a significant impact on color, aroma, and taste [8,27], which were enriched in the fruit of *C. luteoflora*, and similar results were found in the seeds of *C. oleifera* [28]. Flavonoids are known as “plant gold“ and are also abundant in *C. luteoflora*, which have a variety of health effects, such as lowering cholesterol, antioxidant activity, and enhancing immunity. Organic acids contribute to the aroma and flavor of tea [29] and are important determinants of tea quality [30], accounting for 9.0% of *C. luteoflora*. In addition, tea is an important source of polyphenols, it is a compound with strong antioxidant and anti-free-radical effects, and its oxidation products contribute to the color change in tea soup [27]. It has many biological effects such as anti-inflammatory, antioxidant, antitumour, and cardiovascular regulatory effects [31], and its relative content is the highest in flowers. Sugars and alcohols in tea play an important role in regulating tea flavor, reducing the risk of diabetes, and scavenging free radicals [8]. Lipid is one of the important components of tea, accounting for 7.1% in *C. luteoflora*. Its degradation is considered to be one of the main factors responsible for the formation of tea aroma, which is related to the sensory quality of tea [32], and its content is the highest in leaves. Alkaloids are a kind of nitrogen-containing alkaline organic compounds [33], which are the core secondary metabolites that affect the quality of tea. The composition and content of alkaloids largely determine the quality and suitability of tea, which determines the development and utilization value of tea resources [34]. In addition, *C. luteoflora* also contains biologically active metabolites such as ketones, aldehydes, acids, steroids, nucleotides, phenylpropanoids, and vitamins. However, the composition and content of these compounds are different in different organs of *C. luteoflora*, which gives potential application value to different organs of *C. luteoflora*.

Among the DEMs, the types and contents of terpenoids in the root differential metabolites were greater, and 27 of the 58 dominant metabolites were enriched in the roots. Some dominant metabolites such as arachidonic acid, play a key role in inhibiting inflammation [35], Lucidone and Isoliquiritigenin have high anti-tumor efficacy [36,37]. These provide a reference for the development of the medicinal value of roots.

The types and contents of DEMs such as amino acids, sugars and alcohols, organic acids, nucleotides, and uronic acids were highest in the flowers of *C. nitidissima*, which were found in *C. petelotii* [13] and *C. sinensis* [38,39], and it has been reported that these components give the tea flower beverage a unique sweet and umami taste. In Chinese medicine, tea flowers are utilized as deodorants, skin care ingredients, cough suppressants, and cough expectorants [40]. Furthermore, tea flowers are enriched in active ingredients that exhibit antioxidant, anti-inflammatory, immunostimulating, antitumour, hypoglycemic, antiobesity, and antiallergic properties [8]. Among the dominant DEMs, 75 dominant DEMs such as mannose, shikimic acid, D-gluconic acid, and kaempferol are enriched in the flowers. As a monosaccharide, mannose is 100 times less than glucose in the human blood, which is beneficial to human health and can effectively resist inflammation and autoimmune diabetes [41]. As an important natural product in industry, shikimic acid is a critical starting material for the production of the anti-influenza drug oseltamivir phosphate, which has many pharmacological effects such as antibacterial, antiplatelet, and thrombotic activity [42]. In addition, the shikimic acid biosynthesis pathway is considered to be one of the most important pathways in flavonoid biosynthesis, and flavonoids are the main aroma components and functional components in tea. The up-regulation of its key genes increases the concentration of flavonoids and flavonoid glycosides, affecting the taste and quality of white tea [43]. D-gluconic acid is a noncorrosive, nonvolatile, nontoxic, mild organic acid and a natural constituent of fruits, plants, wine, and honey that provides a refreshing sour taste. It is listed as a generally permitted food additive (E574) by the EFSA, and it is listed as a generally recognized as safe (GRAS) additive by the US FDA [44]. Kaempferol is a flavonoid found in a variety of plants. It is a characteristic compound in flowers of *C. sinensis* and has many biological activities in green tea [45]. It improves cardiac function by alleviating myocardial apoptosis, fibrosis, oxidative stress, and inflammation, while preserving mitochondrial function and calcium homeostasis [46].

The fruit of *C. luteoflora* is rich in DEMs such as amino acids, nucleotides, steroids, and vitamins, with a high content of sugars and alcohols and terpenoids, and its nutrients are rich. Liu et al. [22] found that the seed of *C. luteoflora* has a high content of essential amino acids, which provides a basis for the development and application of tea oil of *C. luteoflora*. Among the dominant DEMs, there are 45 dominant DEMs such as pterostilbene, L-ascorbic acid, and riboflavin, etc. Among them, pterostilbene is a phytoalexin for polyphenol compounds, which was originally isolated from the heartwood of red sandalwood and has potential health benefits in inflammatory skin diseases, light protection, cancer prevention and treatment, insulin sensitivity, blood glucose and blood lipid levels, cardiovascular diseases, aging, memory, and cognition [47]. L-ascorbic acid (AsA) is an indispensable compound for human health. As a major antioxidant, AsA can not only maintain redox balance and resist biotic and abiotic stresses, but can also regulate plant growth, induce flowering, and delay senescence through complex signal transduction networks [48]. In the linoleic acid antioxidant test, it was found that green tea polyphenols mixed with vitamin E and vitamin C can synergistically protect lipid peroxidation [49]. This gives the fruit of *C. luteoflora* potential edible and medicinal value.

The stems of *C. luteoflora* are rich in lipids and alkaloids, and lipids are used to regulate the flavor and nutritional quality of fermented foods and are associated with the sensory qualities of tea [32]. Oolong tea made from leaves and stems has a more aromatic smell than leaf-only tea [50]. In addition, the stems are also enriched with high potential medicinal components, 27 dominant DEMs such as salicylic acid, D-(-)-quinic acid, mannitol, (-)-catechin gallate, etc. Similarly, salicylic acid has a high content in the stem epidermis of *C. sinensis*, which is an important immune-related hormone and participates in many plant growth and development processes [51]. Anthracnose is a major leaf disease in tea plants caused by *Colletotrichum gloeosporioides*. During pathogen infection, SA biosynthesis and signal transduction are enhanced in plants, with SA inducing the expression of disease-resistance-related genes to enhance plant disease resistance [52]. Mannitol is a polyol with a similar structure to mannose aldehyde hexose, which is a soluble carbohydrate and widely distributed in tea [53], and can induce JA accumulation and the increase in peroxidase activity to improve the tolerance to osmotic stress in tea plants [54]. The free form of quinic acid is widely present in plants and is accumulated in coffee, tea, and some fruits [55]; its content can be used as an indicator to predict the quality of green tea [56]. As one of the metabolites of tea catechins, (-)-catechin gallate has various beneficial effects on human health due to its antioxidant activity, including scavenging free radicals, metal chelation, and inhibiting lipid peroxidation [57], and it has a more effective cholesterol-lowering effect than epigallocatechin gallate and epicatechin gallate [58].

Among the DEMs, the types and contents of terpenoids, flavonoids, polyphenols, alkaloids, phenylpropanoids, quinones, and coumarins were the highest in the leaves of *C. luteoflora*, and similar results were reported in Theaceae plants [10,11,30]. Among the dominant DEMs, 119 metabolites such as oleanolic acid, L-Glucose, D-Arabitol, and Eugenol are enriched in the leaves. Oleanolic acid as a pentacyclic triterpenoid compound from a variety of biologically active plant sources; it has attracted great attention from the scientific community due to its biological activity against various diseases. It can effectively combat dyslipidemia, diabetes, and metabolic syndrome by enhancing the insulin response, maintaining β cell function and survival, and preventing diabetic complications [59]. Similarly, L-Glucose and D-Arabiitol, as the main components of tea polysaccharides, have the effects of lowering blood glucose, antioxidation, lowering blood lipids, and enhancing immunity [60], while D-Arabiitol is widely used as a low-calorie sweetener in the food industry [61]. In addition, Eugenol is a natural phenolic compound with antibacterial, anti-inflammatory, and anti-tumor effects [62], and it is the main component of clove essential oil, which helps to contribute to the taste of white tea, green tea, and oolong tea. It is famous for its ability to add floral fragrance to tea plants and promote human health, and plays a signal role in the regulation of cold and drought tolerance of *C. japonica* [63]. It is worth mentioning that the types and levels of metabolites of plants will fluctuate greatly in different seasons, growth stages, and environments. Therefore, the types and contents of metabolites in different organs of *C. luteoflora* at different growth stages need to be further studied.

In the study of the components of *C. luteoflora*, previous researchers only detected the volatile components of its flowers and leaves, as well as the amino acids in the fruit. However, we comprehensively analyzed the components and contents of the five organs of *C. luteoflora*, including the roots, flowers, fruits, stems, and leaves. The metabolites and contents of each organ and the DMEs were obtained, which was helpful for a more comprehensive understanding and utilization of *C. luteoflora*.

## 5. Conclusions

In this study, LC–MS was used to detect the five organs of *C. luteoflora*, including the roots, flowers, fruits, stems, and leaves. The types and contents of metabolites in each organ were systematically analyzed. The results showed that 815 metabolites were detected in the roots, stems, leaves, flowers, and fruits of *C. luteoflora*, and they were rich in chemical components. These metabolites were mainly divided into 18 main categories, including terpenoids (17.1%), amino acids (10.4%), flavonoids (10.3%), sugars and alcohols (9.4%), organic acids (9.0%), lipids (7.1%), polyphenols (6.1%), alkaloids (4.9%), etc. A total of 684 DEMs were identified in five organs, mainly including terpenoids, amino acids, flavonoids, sugars and alcohols, and organic acids, which were annotated into 217 KEGG metabolic pathways, among which metabolic pathways, ABC transporters, the biosynthesis of cofactors, and the biosynthesis of amino acids were significantly enriched. Among them, flowers are rich in flavonoids, polyphenols, organic acids, and steroids; fruits are rich in amino acids, alkaloids, vitamins, and xanthones; stems are rich in lignans; and leaves have the highest relative content of phenylpropanoids, ketoaldehydic acids, quinones, sugars and alcohols, terpenoids, coumarins, lipids, and others; meanwhile, the metabolite content is lower in the roots. Different organs were enriched with different metabolites, among which there are eight common DEGs such as Scytophycin B, Azukisaponin VI, Meliasenin E, beta-Citraurin, Floctafenine, cis-1,2-Dihydroxy-4-methylcyclohexa-3,5-diene-1-carboxylate, Aspartylglycosamine, and Sulfate. Among the dominant DEMs, 58 were in the roots, including arachidonic acid, lucidone, isoliquiritigenin, etc.; 75 were in the flowers, including mannose, shikimic acid, d-gluconic acid, kaempferol, etc.; 45 were in the fruit, including pterostilbene, l-ascorbic acid, riboflavin, et; 27 were in the stems, including salicylic acid, d-(-)-quinic acid, mannitol, (-)-catechin gallate, etc.; There are a maximum number of 119 dominant metabolites in the leaves, including oleanolic acid, l-glucose, d-arabitol, eugenol, etc. The various organs of *C. luteoflora* contain many bioactive metabolites, which are significantly different. These provide a theoretical reference for further understanding the chemical composition of *C. luteoflora* and the development of its tea, flower tea, edible oil, health care products, and medicinal components.

## Figures and Tables

**Table 1:** Relative contents of the root, flower, fruit, stem, and leaf metabolites of *C. luteoflora*.

Metabolites	Flavonoids	Polyphenols	Organic Acid	Steroids	Nucleotides	Amino Acids	Alkaloids	Vitamins	Xanthones	Lignans	Phenylpropanoids	Ketones, Aldehydes, Acids	Quinones	Sugars and Alcohols	Terpenoids	Coumarins	Lipid	Others
Roots	13.83 × 10^7^ ± 8.46 × 10^7^ c	6.43 × 10^6^ ± 1.71 × 10^6^ c	27.32 × 10^7^ ± 4.65 × 10^7^ d	9.07 × 10^5^ ± 3.07 × 10^4^ b	2.97 × 10^6^ ±5.43 × 10^5^ b	12.85 × 10^7^ ± 6.52 × 10^7^ a	4.41 × 10^6^ ± 0.99 × 10^6^ bc	3.03 × 10^5^ ± 5.71 × 10^4^ b	—	3.00 × 10^4^ ± 0.61 × 10^4^ b	7.13 × 10^6^ ± 4.12 × 10^6^ c	1.39 × 10^6^ ± 0.46 × 10^5^ d	7.58 × 10^6^ ± 5.35 × 10^6^ bc	36.44 × 10^7^ ± 0.51 × 10^7^ c	1.76 × 10^7^ ± 1.75 × 10^6^ c	1.17 × 10^6^ ± 1.73 × 10^5^ c	1.76 × 10^7^ ± 4.84 × 10^6^ c	8.80 × 10^7^ ± 8.33 × 10^6^ a
Flowers	46.56 × 10^7^ ± 5.10 × 10^7^ a	58.83 × 10^6^ ± 2.62 × 10^6^ a	10.36 × 10^8^ ± 5.39 × 10^7^ a	3.55 × 10^6^ ± 3.06 × 10^6^ a	7.84 × 10^6^ ± 1.64 × 10^6^ a	11.48 × 10^7^ ± 4.13 × 10^6^ a	11.41 × 10^6^ ± 1.26 × 10^6^ b	2.09 × 10^6^ ± 1.03 × 10^6^ b	—	1.02 × 10^4^ ± 0.29 × 10^4^ b	2.09 × 10^7^ ± 4.78 × 10^6^ b	6.04 × 10^6^ ± 5.71 × 10^5^ cb	15.81 × 10^6^ ± 1.77 × 10^6^ b	65.27 × 10^7^ ± 5.47 × 10^7^ a	1.75 × 10^7^ ± 7.54 × 10^6^ c	5.88 × 10^5^ ± 2.03 × 10^5^ c	8.65 × 10^7^ ± 1.58 × 10^7^ b	5.27 × 10^7^ ± 4.23 × 10^6^ b
Fruits	4.78 × 10^7^ ± 2.06 × 10^7^ c	3.38 × 10^6^ ± 0.19 × 10^6^ c	42.96 × 10^7^ ± 8.44 × 10^7^ c	3.34 × 10^5^ ± 7.35 × 10^4^ b	6.79 × 10^6^ ± 6.06 × 10^5^ a	15.35 × 10^7^ ± 2.92 × 10^7^ a	23.58 × 10^6^ ± 9.24 × 10^6^ a	20.89 × 10^6^ ± 4.23 × 10^6^ a	0.13 × 10^4^ ± 0.12 × 10^4^ a	5.57 × 10^4^ ± 2.74 × 10^4^ ab	2.65 × 10^6^ ± 9.66 × 10^5^ c	1.40 × 10^6^ ± 1.88 × 10^5^ cd	2.35 × 10^6^ ± 4.89 × 10^5^ c	49.10 × 10^7^ ± 4.35 × 10^7^ b	9.68 × 10^6^ ± 1.00 × 10^6^ c	1.03 × 10^5^ ± 2.77 × 10^4^ c	2.12 × 10^7^ ± 2.40 × 10^6^ c	2.64 × 10^7^ ± 3.13 × 10^6^ c
Stems	27.24 × 10^7^ ± 4.95 × 10^7^ b	21.74 × 10^6^ ± 5.28 × 10^6^ b	74.07 × 10^7^ ± 15.12 × 10^7^ b	7.26 × 10^5^ ± 2.96 × 10^5^ b	1.72 × 10^6^ ± 1.49 × 10^5^ b	11.08 × 10^7^ ± 3.02 × 10^7^ a	3.95 × 10^6^ ± 0.59 × 10^6^ bc	6.55 × 10^5^ ±3.54 × 10^4^ b	—	8.08 × 10^4^ ± 3.29 × 10^4^ a	2.55 × 10^7^ ± 2.96 × 10^6^ b	2.40 × 10^6^ ± 6.03 × 10^5^ c	7.68 × 10^6^ ± 5.79 × 10^6^ bc	28.79 × 10^7^ ± 2.52 × 10^7^ c	5.46 × 10^7^ ± 5.16 × 10^6^ b	6.98 × 10^6^ ± 4.68 × 10^5^ b	2.36 × 10^7^ ± 5.22 × 10^6^ c	5.70 × 10^7^ ± 7.23 × 10^6^ b
Leaves	31.38 × 10^7^ ± 4.41 × 10^7^ cb	25.39 × 10^6^ ± 1.11 × 10^6^ b	37.94 × 10^7^ ± 4.27 × 10^7^ cd	6.20 × 10^5^ ± 1.52 × 10^5^ b	1.49 × 10^6^ ± 3.11 × 10^5^ b	2.67 × 10^7^ ± 2.17 × 10^6^ b	2.37 × 10^6^ ± 0.05 × 10^6^ c	6.50 × 10^5^ ± 8.88 × 10^4^ b	—	6.54 × 10^4^ ± 1.56 × 10^4^ ab	11.50 × 10^7^ ± 1.02 × 10^7^ a	10.10 × 10^6^ ± 5.35 × 10^6^ a	7.72 × 10^7^ ± 8.26 × 10^6^ a	71.56 × 10^7^ ± 6.57 × 10^7^ a	6.48 × 10^7^ ± 5.50 × 10^6^ a	1.17 × 10^7^ ± 1.46 × 10^6^ a	1.22 × 10^8^ ± 9.14 × 10^6^ a	9.26 × 10^7^ ± 1.59 × 10^7^ a

Note: Different lowercase letters in the same row indicate significant differences (*p* < 0.05); “—” indicates that the metabolite contents in the organs did not reach the quantification line.

## Data Availability

The original contributions presented in the study are included in the article/Supplementary Material. Further inquiries can be directed to the corresponding authors.

## Supplementary Materials

The following supporting information can be downloaded at: https://www.mdpi.com/article/10.3390/molecules29194754/s1, Figure S1: The score chart of OPLS-DA. Figure S2: Heat map of DEMs of the R, F, Fr, S and L of *C. luteoflora*. Figure S3: Total ion chromatogram of *C. luteoflora* under negative ion mode (A) and positive ion mode (B). Table S1: The metabolites identified and quantified in *C. luteoflora*. Table S2: The DEMs identified and quantified in *C. luteoflora*. Table S3: Up- and down-regulated metabolites in R, F, Fr, S and L of *C. luteoflora*.
