# Supplementary material for: Widely Targeted Metabolomics Analysis of the Roots, Stems, Leaves, Flowers, and Fruits of Camellia luteoflora, a Species with an Extremely Small Population
Source: Molecules. 2024 Oct 8;29(19):4754. doi: 10.3390/molecules29194754 (PMC11477736; doi:10.3390/molecules29194754)
Supplement: Supplementary file 1 [file molecules-29-04754-s001.zip › Figure S1.pdf]

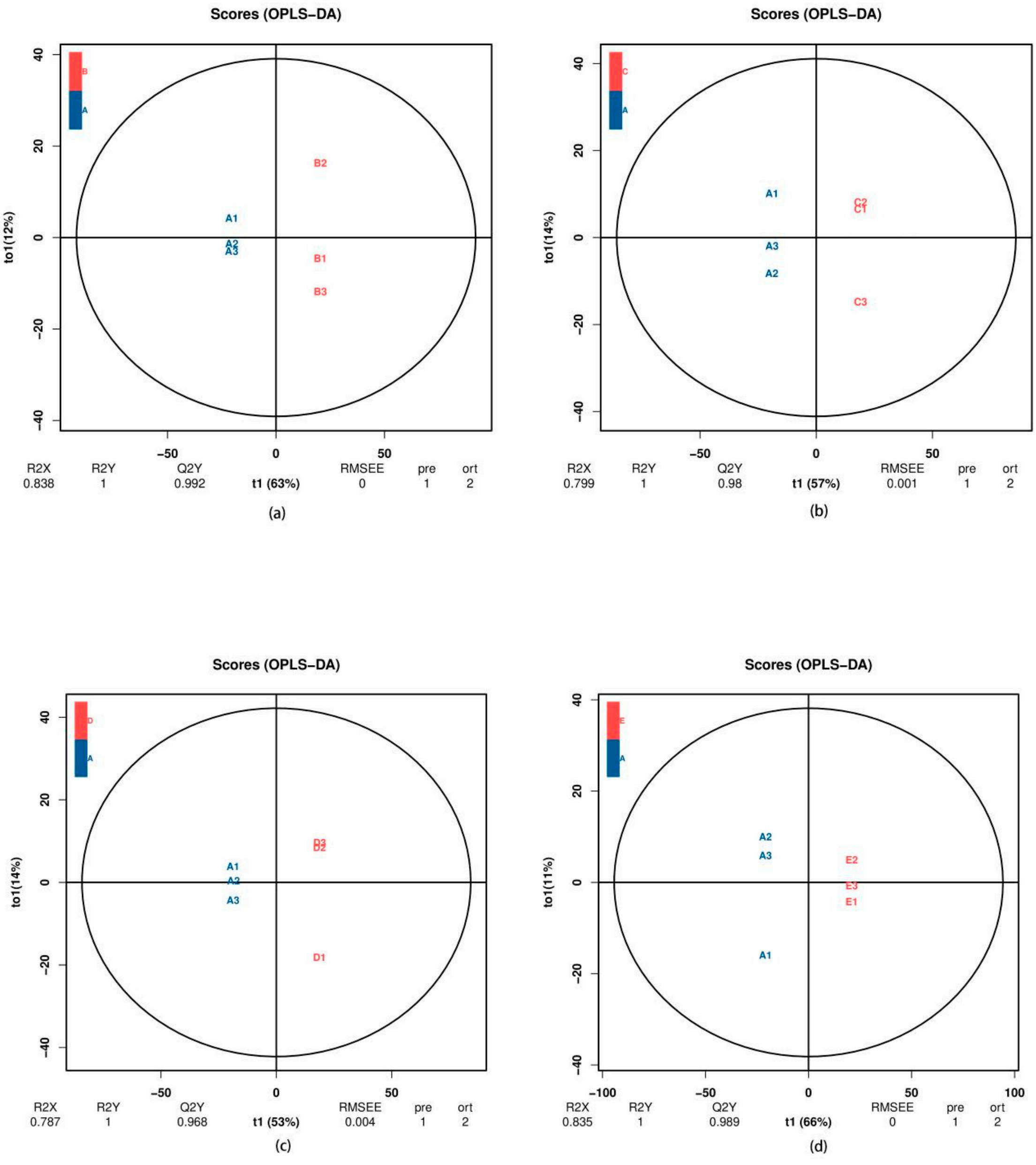
Scores (OPLS−DA)
to1(12%)
t1 (63%)
R2X R2Y Q2Y RMSEE pre ort
0.838 1 0.992 0 1 2
(a)
Scores (OPLS−DA)
to1(14%)
t1 (57%)
R2X R2Y Q2Y RMSEE pre ort
0.799 1 0.98 0.001 1 2
(b)
Scores (OPLS−DA)
to1(14%)
t1 (53%)
R2X R2Y Q2Y RMSEE pre ort
0.787 1 0.968 0.004 1 2
(c)
Scores (OPLS−DA)
to1(11%)
t1 (66%)
R2X R2Y Q2Y RMSEE pre ort
0.835 1 0.989 0 1 2
(d)

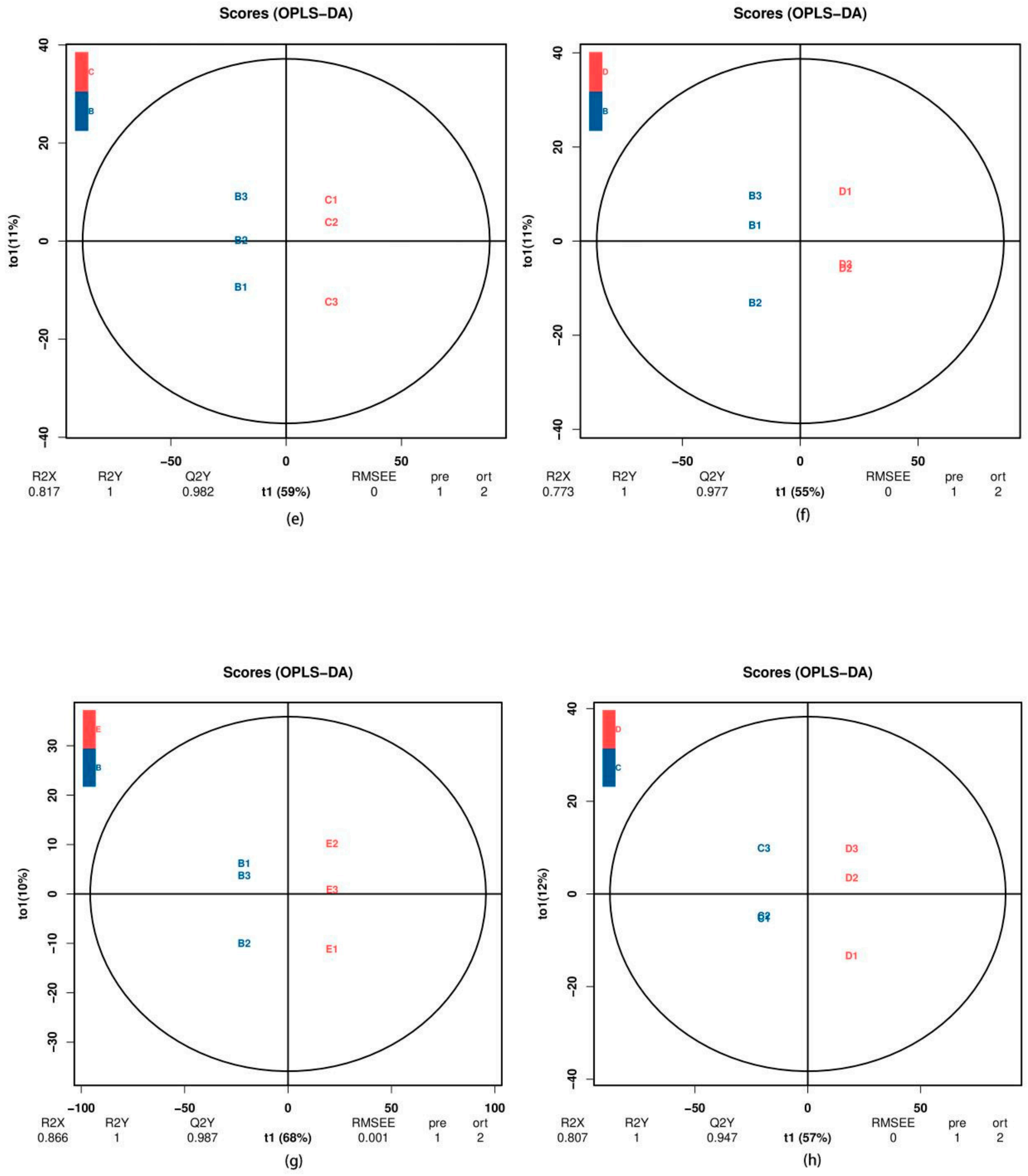

Scores (OPLS−DA)
C
B
B3
C1
C2
B2
B1
C3
to1(11%)
R2X 0.817
R2Y 1
Q2Y 0.982
t1 (59%)
RMSEE 0
pre 1
ort 2
(e)
Scores (OPLS−DA)
D
B
B3
D1
B1
D3
D2
B2
to1(11%)
R2X 0.773
R2Y 1
Q2Y 0.977
t1 (55%)
RMSEE 0
pre 1
ort 2
(f)
Scores (OPLS−DA)
E
B
E2
B1
B3
E3
B2
E1
to1(10%)
R2X 0.866
R2Y 1
Q2Y 0.987
t1 (68%)
RMSEE 0.001
pre 1
ort 2
(g)
Scores (OPLS−DA)
D
C
C3
D3
D2
D1
to1(12%)
R2X 0.807
R2Y 1
Q2Y 0.947
t1 (57%)
RMSEE 0
pre 1
ort 2
(h)

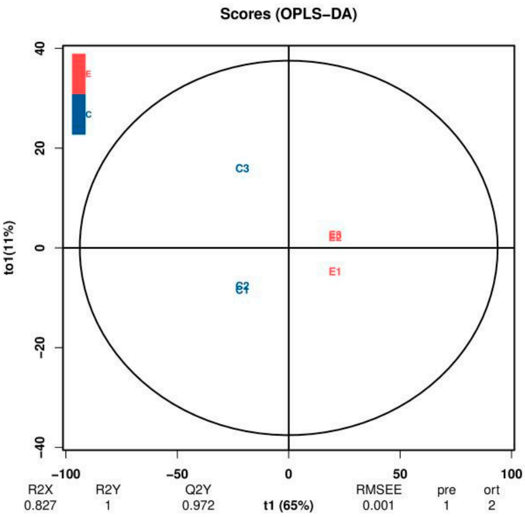

(i)

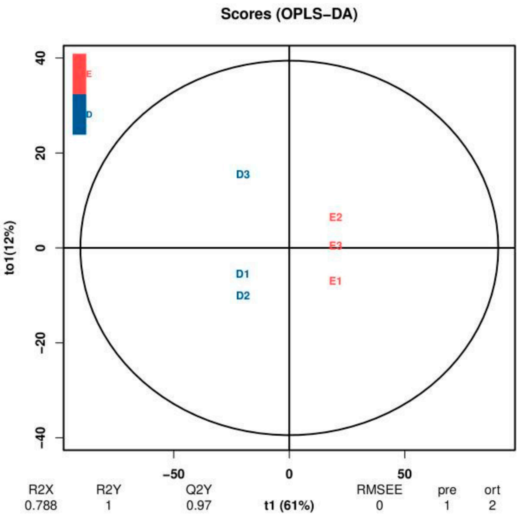

(j)

OPLS-DA score chart

R.vs..F (R2X=0.838, R2Y=1, Q2Y=0.992); R.vs..Fr (R2X=0.799, R2Y=1, Q2Y=0.98)

R.vs..S (R2X=0.787, R2Y=1, Q2Y=0.968); R.vs..L (R2X=0.835, R2Y=1, Q2Y=0.989)

F.vs..Fr (R2X=0.817, R2Y=1, Q2Y=0.982); F.vs..S (R2X=0.773, R2Y=1, Q2Y=0.977)

F.vs..L (R2X=0.866, R2Y=1, Q2Y=0.987); Fr.vs..S (R2X=0.807, R2Y=1, Q2Y=0.947)

Fr.vs..L (R2X=0.827, R2Y=1, Q2Y=0.972); S.vs..L (R2X=0.788, R2Y=1, Q2Y=0.97)
