# Supplementary material for: Widely Targeted Metabolomics Analysis of the Roots, Stems, Leaves, Flowers, and Fruits of Camellia luteoflora, a Species with an Extremely Small Population
Source: Molecules. 2024 Oct 8;29(19):4754. doi: 10.3390/molecules29194754 (PMC11477736; doi:10.3390/molecules29194754)
Supplement: Supplementary file 1 [file molecules-29-04754-s001.zip › Figure S2.pdf]

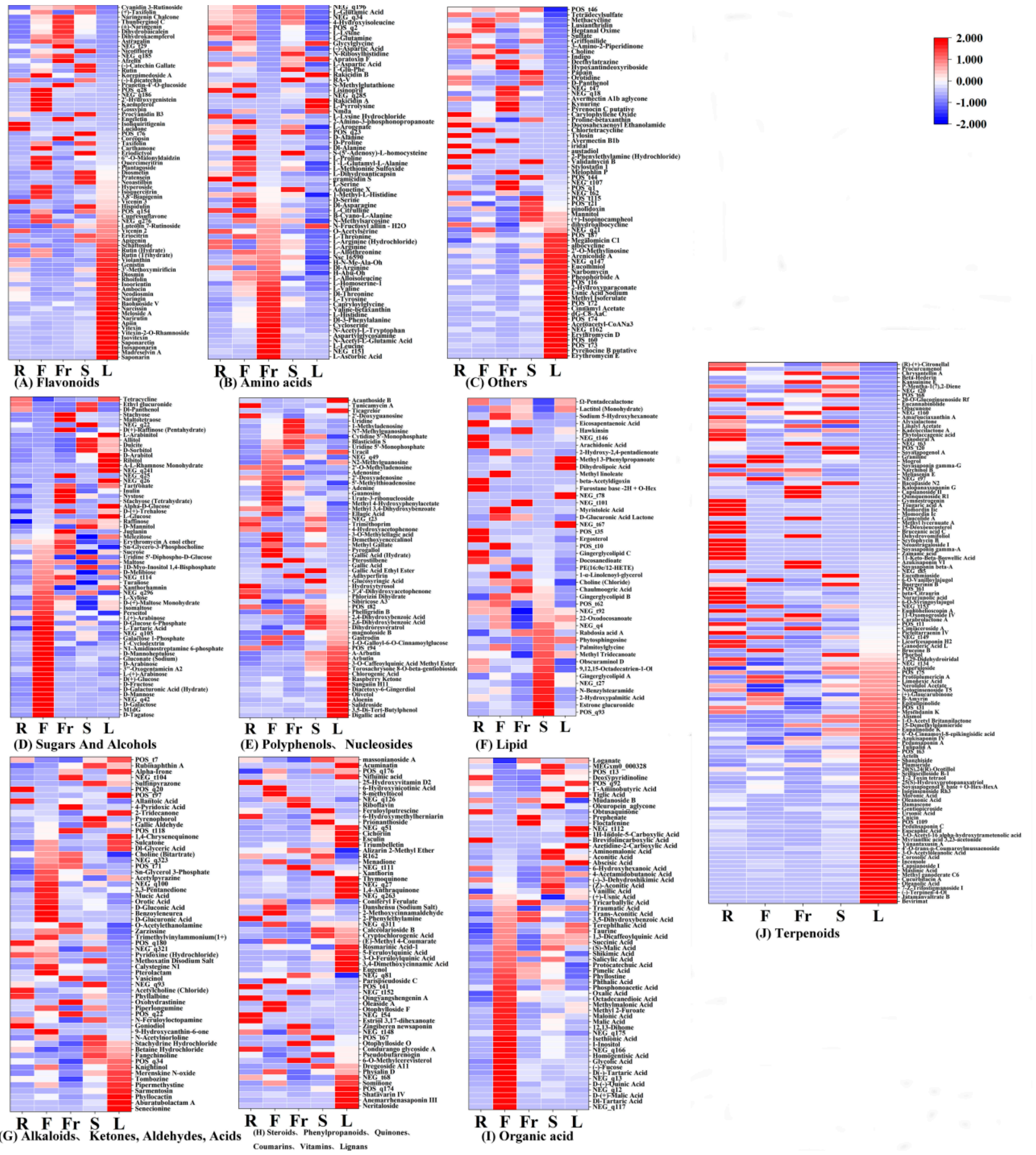

DEMs of R, F, Fr, S and L

(A) Flavonoids (B) Amino Acids (C) Others (D) Sugars and Alcohols (E) Polyphenols, Nucleotides (F) Lipid (G) Alkaloids, Ketones, Aldehydes, and Acids (H) Steroids, Phenylpropanoids, Quinones, Coumarins, Vitamins, Lignans (I) Organic Acids (J) Terpenoids
