# Supplementary figures and images for: Widely Targeted Metabolomics Analysis of the Roots, Stems, Leaves, Flowers, and Fruits of Camellia luteoflora, a Species with an Extremely Small Population
Source: Molecules. 2024 Oct 8;29(19):4754. doi: 10.3390/molecules29194754 (PMC11477736; doi:10.3390/molecules29194754)

A

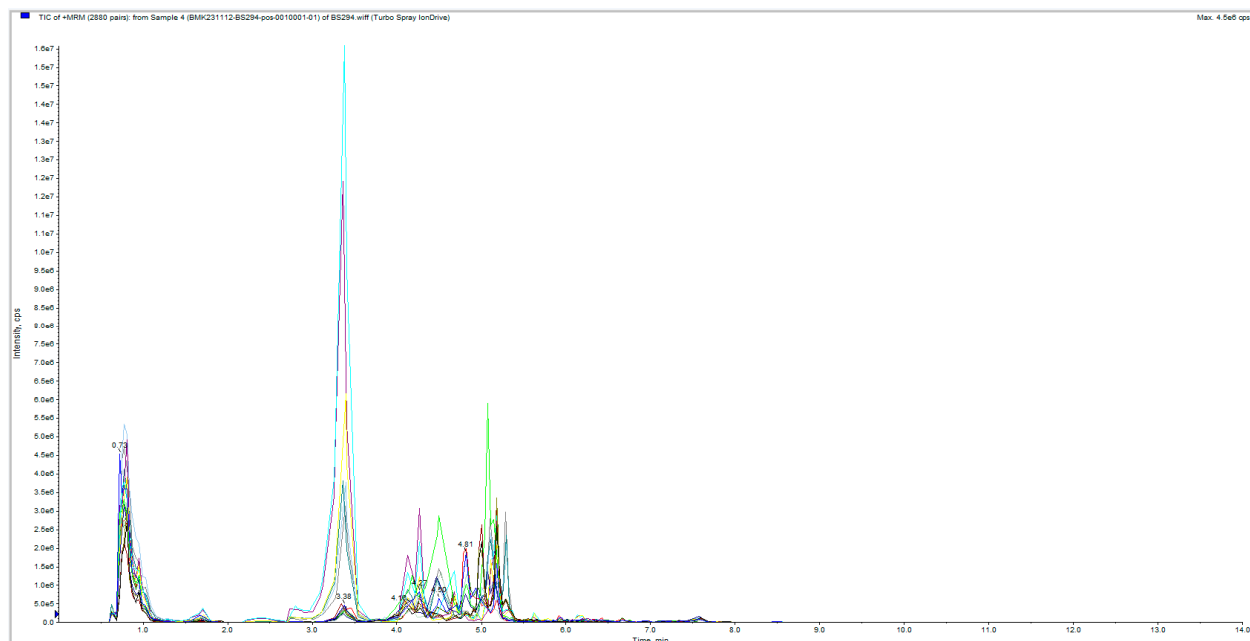

B

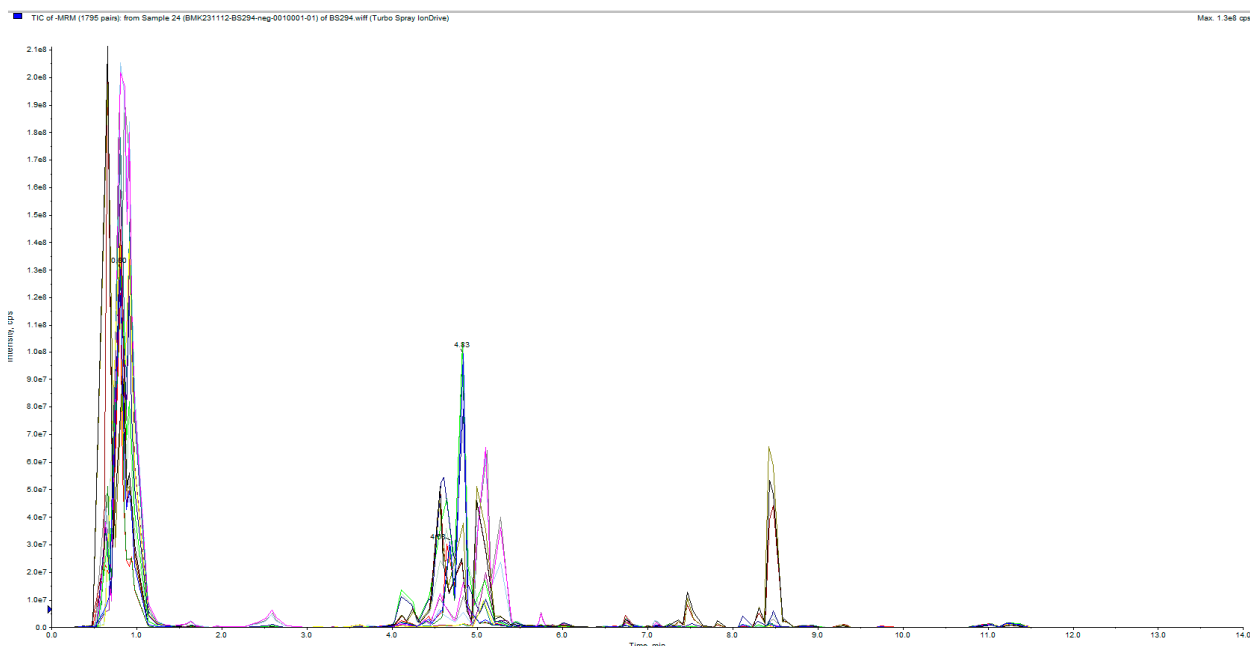

Sample mass spectrometry analysis of positive ion(A) and negative ion(B) flow chart

Supplement: Supplementary file 1 [file molecules-29-04754-s001.zip › Figure S3.pdf]
